# *The Organon* and Materia Medica Club of the Bay Cities of California

**Published:** 1896-05

**Authors:** W. E. Ledyard

**Affiliations:** Secretary


Reported by Eleanor F. Martin, M. D.

---

### *THE ORGANON* AND MATERIA MEDICA CLUB OF THE BAY CITIES OF CALIFORNIA.

The regular semi-monthly meeting was held at the office of Dr. J. M. Selfridge, in Oakland, Friday evening, December 6th, 1895.

Members present: Drs. J. M. Selfridge, A. McNeil, M. T.

Wilson, W. E. Ledyard, E. W. Bradley, G. J. Augur, and George H. Martin. Visitor, Mr. A. Johnson.

The meeting was called to order by the President, Dr. J. M. Selfridge, at 8.15 o'clock. The minutes of the previous meeting were read by the Secretary, and after slight corrections by Drs. Augur and Ledyard, were approved.

Dr. Selfridge stated that he had revised his paper, "Infinitesimals in Nature," and read selections from it, whereupon Dr. Wilson moved that the paper be made a part of the minutes, and that it be published in THE HOMŒOPATHIC PHYSICIAN. (See February Number, p. 68.) Seconded by Dr. Chapman, and carried.

The President then appointed Dr. McNeil reader of the evening, who commenced *The Organon* at Sections 8 and 9.

### DISCUSSION.

Dr. McNeil—These sections are upon the theory of vital force. There is a large body of old-school physicians who maintain the theory of vital force. It is necessary to have the "vital force" in order to account for sickness. Neither physics nor chemistry will do it.

Sections 10 and 11 were then read.

### DISCUSSION.

Dr. McNeil—The old-school physicians are always speaking of the pathological changes taking place. There is a time, however, before the pathological changes occur. If they are the causes of disease the patient is really well until these changes take place. We know that a man may be sick some time before any pathological change takes place.

Dr. Ledyard—The old-school men mistake the effect for the cause.

Dr. McNeil—They are all the time endeavoring to remove the *product* of the disease and not the *cause*, consequently they do not remove the disease at all.

Dr. Martin—I think Dr. McNeil goes a little too far in his last statement. The allopathic physician who does as he says is extremely superficial. Gowers, in the treatment or analysis of disease, first starts in with the causes, then the pathological anatomy, the symptomatology, the pathology, the differential diagnosis, and the prognosis. Pathology is a discourse upon disease, or the analysis of symptoms and causes. *Worry* will produce a disease or symptoms that will indicate a disease. Neurologists try to find a drug that will act pathologically. They try to prescribe, however, for the cause of the trouble as well as for the pathological condition.

Dr. McNeil—Upon this point I will acknowledge that the neurologist is more likely to do as Dr. Martin states than any of the others. The neurologist comes nearer to the cause of disease. He sees more clearly than the others.

Dr. Ledyard—However that may be in regard to their scientific explanations, etc., their treatment is to remove the pathological condition. They remove tumors, etc. They remove fæces in constipation and think they have cured. Their treatment is directed against the *result* rather than against the *cause* of disease.

Section 12 was then read.

### Discussion.

Dr. Selfridge—Hahnemann does not want us to try to find out what the vital force is.

Dr. McNeil—He evidently is striking here at one of the follies of the old school, and that is that they are always dabbling in *speculations.* Hahnemann wants us to take certain things for granted, and leave speculation alone.

Sections 13–16 were then read.

### Discussion.

Dr. McNeil—It is quite clear that a great many mistakes have been made as regards therapeutics, not only among the laity, but in the old school. In reading the life of Hahnemann, it can be seen that in the science of that day there was a great deal more materialism than there is to-day. They used to think that disease was something to be chased out, sweated out, etc., and some of them have not gotten over it yet.

Section 17 was next read.

### Discussion.

Dr. Chapman—This does not look as if Hahnemann was an atheist, as some have said of him. Judging from the notes to this Section, he seems to have been a devoutly pious man.

Dr. McNeil—That first note is a striking example of showing the immateriality of disease.

Dr. Martin—I have seen that enacted once. In the Hawaiian Islands there is a custom of praying people to death. A man forty years of age dropped dead in front of my house. I went out and picked him up. The autopsy revealed heart disease. What really caused his death was the fact that upon that morning he had heard that for three weeks enemies had been praying him to death, and he died at the very hour that they wanted him to die.

Dr. Selfridge—I can conceive of one being frightened to death, but I should look at that case with suspicion, a diseased condition really being present.

Dr. McNeil—We know that witch-craft was the source of sickness and death in old times. This suggests the man Schlatter, who is at present making so many remarkable cures, and I do not doubt that he is doing so. The people have become thoroughly convinced that there is a divine power in him, and it works a cure with their nervous systems. All over the world there are shrines, as St. Anne's, Lourdes, etc., where people go to be cured, and I believe there are cures in this way for certain people.

Dr. Martin—There is another side to that question. One of the daily papers said that Schlatter's cures were not only due to the faith of the people, but to the confidence he has in himself. Let us once make up our minds that the remedy we prescribe is the correct one, and it has a great deal to do with the cure.

Dr. McNeil—It is the confidence in ourselves, or we inspire our patients with confidence and we make a good selection in our remedy. I believe that Schlatter is an impostor. He may be honest enough in what he intends to do, but he certainly has no divine power.

Dr. Chapman—Faith will not touch pathological states.

Dr. McNeil—I believe that it is only in functional diseases that cures are made by Schlatter. Bernheim on hypnotism claims that all these cures are cures by *suggestion*, without any supernatural power, and they are all functional diseases that can be cured in this way.

Dr. Selfridge—I do not think that I can agree with Dr. Martin, that any man's belief in his own medicine will effect a cure. I have cured when I was not sure of my remedy.

Dr. Ledyard—I heard that *Pulsatilla* would change the position of the child in the uterus. I had a case of shoulder presentation which became more fixed with every pain. I gave a little powder of *Pulsatilla*$^{200}$, and while my finger was on the shoulder of the child it turned, and I gave the credit to the *Pulsatilla.*

Dr. Martin—I do not wish to infer that the confidence alone in the medicine would cure.

Section 18 was read.

Upon motion of Dr. Wilson, seconded by Dr. McNeil, the meeting then adjourned, to meet at the office of Dr. W. E. Ledyard, 233 Post Street, San Francisco, the third Friday in December, when the reading of *The Organon* would be commenced at Section 19.

W. E. LEDYARD, *Secretary.*

